# Enhanced Dielectric Environment Sensitivity of Surface Plasmon-Polariton in the Surface-Barrier Heterostructures Based on Corrugated Thin Metal Films with Quasi-Anticorrelated Interfaces

**DOI:** 10.1186/s11671-017-1974-3

**Published:** 2017-03-23

**Authors:** Alexander V. Korovin, Nicolas L. Dmitruk, Sergii V. Mamykin, Viktor I. Myn’ko, Mariya V. Sosnova

**Affiliations:** grid.466789.2 V.Ye. Lashkaryov Institute of Semiconductor Physics, NAS of Ukraine, 41 prospect Nauki, Kiev, 03028 Ukraine

**Keywords:** Surface plasmon-polaritons, Chalcogenide, Sensors, Signal sensitivity, Polarization sensitivity

## Abstract

A new approach to the formation of a 1D planar periodicity on the front of a plasmonic photodetector based on Schottky barrier is proposed. It allows forming a 1D planar periodicity with corrugation at the “metal/environment” interface by laser interference lithography using embedded chalcogenide wires, whereas the “metal/semiconductor” interface is flat that leads to reducing of surface recombination losses at Shottky barrier in contrary to the conventional technology for forming corrugated metal films on the semiconductor surface requiring chemical etching of the semiconductor substrate. In this case, the metal film interfaces are quasi-anticorrelated as opposed to correlated ones in the conventional technology. It has been theoretically predicted that the polarization sensitivity (*T*_*p*_/*T*_*s*_) strongly depends on the cross-sectional shape of chalcogenide wires and reaches a value of 8. Furthermore, it was theoretically found that the maximum sensitivity of the signal intensity on the environment refractive index is three times larger than for an equivalent structure obtained by conventional technology. Comparison of experimental data for the photocurrent in the case of two types of correlation between metal film interfaces demonstrates good agreement with numerical simulations.

## Background

One of the implementations of high-sensitive active sensors is based on the grating coupler effect for the excitation of surface plasmon-polaritons (SPP) [1] in the surface-barrier heterostructures with Schottky barrier [2, 3]. Today, there are several techniques of forming a diffraction grating on the semiconductor substrate, namely, the laser-induced periodic surface structures [4], extreme ultraviolet lithography [5], electron beam lithography [6], ion beam lithography [7], nanoimprint technology [8], pulsed laser interference lithography [9], and laser interference lithography [10, 11]. The latter is well known and relatively cheap method that allows to form a planar periodicity on large areas. Unfortunately, the etching of the semiconductor front interface is necessary in this method which leads to additional surface recombination in Schottky barrier [12]. Therefore, it is important that the interface “metal/semiconductor” remains flat in order to reduce the surface recombination losses in the case of plasmon-polariton photodetector based on Schottky barrier. At the same time, it is also necessary to satisfy the condition for resonant excitation of SPP, which can be implemented due to planar periodicity in the case of periodically profiled “metal/environment” interface. Recently, it has been theoretically predicted in [13] that the geometric interrelation between the profiled interfaces of a corrugated metal film increases the coupling between SPPs excited at the opposite interfaces of the metal film with the increasing of light transmittance into the semiconductor active region and the transformation of SPP peak shape from Lorenz type for films with correlated interfaces to Fano type [14] for anticorrelated interfaces.

In this work, we analyze the SPP sensitivity of surface-barrier heterostructures based on Schottky barrier with a periodically corrugated thin metal film with quasi-anticorrelated interfaces, where the diffraction grating is formed only on the external interface of the metal film by inclusion of periodic array of chalcogenide wires, while the “metal/semiconductor” interface is flat. On the one hand, a periodical metal film profiling leads to SPP excitation with Fano peak shape in the transmittance (photocurrent). On the other hand, a flat “metal/semiconductor” interface substantially reduces the surface recombination losses unlike the conventional technology [11]. Thus, the main advantages of such heterostructures are (i) the reducing of surface recombination losses that improves the photodetector properties in the case of the application of these heterostructures as active plasmonic sensors and (ii) the enhancing of the sensitivity of signal intensity in the spectral region near falling down side of Fano-like SPP resonance peak.

## Methods

### Simulations

The principle of operation for the surface plasmon-polariton sensors is based on detection of spectral or angular peculiarities appearing in the optical response due to the effect of SPP excitation [15, 16]. SPP is a quasiparticle resulting from the strong interaction between surface plasmons and photons. Thus, the SPP is a TM-type surface electromagnetic wave localized at the interface of the two media satisfying to the condition of active interface: *ε*_1_<−*R* *e*(*ε*_2_) [17], where *ε*_1_ and *ε*_2_ are the dielectric permittivities of the two contacting media. The main advantage of SPP for the sensor application is the high sensitivity to changes in the optical properties of contacting media [18]. In the case of a flat interface, the SPP wave vector is expressed analytically in the following form: $k_{SPP} = ({\omega /c}) \sqrt {{\varepsilon _{1} \varepsilon _{2}}/ {\left ({\varepsilon _{1} +\varepsilon _{2}} \right)}}$, and this value is larger than all wave vectors of the plane waves in contacting media [17]. Therefore, an addition coupler is needed to excite SPP by the plane wave. The grating coupler is one of the widespread couplers for SPP excitation by the plane wave with a relatively compact implementation [19].

In the case of 1D grating coupler (multilayer structures with 1D planar periodicity), the SPP are excited only in the case of *p*-polarized (transverse magnetic) incident plane wave when the electric field and the reciprocal vector of planar periodicity lie in the plane of incidence. And *s*-polarization (transverse electric) is determined for a configuration in which the electric field is perpendicular to the reciprocal vector and the plane of incidence, where the reciprocal vector of 1D planar periodicity lies in the plane of incidence as well. The position of SPP for 1D planar periodicity depends on the grating period, *L*, and corresponds to the condition of the momentum conservation law (the wave vectors matching) [17]:

1 $$  k_{SPP} = m(2\pi /L)+(2\pi /\lambda)\sin \theta,  $$

where *m* is the diffraction order (*m*=±1, ±2,…), *θ* is the angle of incidence, and *λ* is the wavelength of light in a vacuum.

**Fig. 1:**
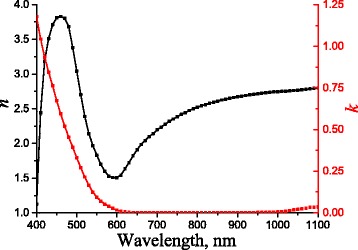
Experimental spectral dependencies of optical constants: refractive index, *n* (*black line*), and exctinction coefficient, *k* (*red line*), for chalcogenide photoresist (As_40_S_30_Se_30_) film

The presence of peculiarities in the transmittance of the heterostructures considered in the present work is associated with SPP excitation at the “metal/environment” interface in the (−1) order of diffraction. The simulations of the light propagation through multilayer corrugated structure with 1D planar periodicity are based on the curvilinear coordinate transformation method in the framework of the differential formalism [20]. All results were obtained in the assumption that the media forming multilayer structure with curvilinear interfaces are homogeneous with bulk optical constants. The optical constants of gold and GaAs were taken from [21] and [22], respectively. For the chalcogenide-based photoresist (ChP) used for periodic structure fabrication, the optical constants were obtained from analysis of the reflectance and transmittance spectra for polarized light from flat satellite samples of corresponding chalcogenide films on transparent glass. Obtained experimental spectra of the optical constants (*n*, *k*) are shown in Fig. 1. Moreover, the residue of ChP in the final device is surrounded by gold with a thickness of about or more than 20 nm, which is around the skin depth for gold. Thus, the impact of ChP at the final stage of SPP is significantly minimized.

To clarify the resonance properties, we use the polarization sensitivity as the ratio between transmittance for *p*- and *s*-polarized light, *T*_*s*,*p*_:

2 $$  S_{p} \equiv \frac{T_{p}}{T_{s}}.  $$

The refractive index sensitivity in the regime of signal detection at the slope of the resonance peak can be estimated in the following form:

3 $$  \frac{\partial I}{\partial n}\equiv \frac{\partial T}{\partial n}\approx \frac{\partial T}{\partial \lambda}\frac{\partial \lambda}{\partial n},  $$

where *∂* *λ*/*∂* *n* is the rate of change of the resonance wavelength position due to changing the environment refractive index and *∂* *T*/*∂* *λ* is the slope of the resonance (signal intensity sensitivity on the wavelength).

Furthermore, to determine the nature of SPP in the case of complicated plasmonic multilayer structures with quasi-anticorrelated interfaces, the spatial distribution of the electric field intensity in the plane of incidence is used in the work.

### Sample Preparation

The quasi-anticorrelated interfaces of plasmon-carrying film on the flat semiconductor substrate forming Schottky barrier were produced by laser interference lithography method [11] with the following technological stages: (i) layer-by-layer vacuum deposition of a thin metal film (Au) and a vitreous chalcogenide photoresist (As_40_S_30_Se_30_) film on a flat semiconductor substrate (GaAs); (ii) the exposure of this pattern by the interference field of two beams of He-Ne (*λ*=488 nm) lasers with period 200–800 nm; (iii) the subsequent chemical etching to form the 1D periodic array of chalcogenide wires on the surface of a flat Au film; (iv) the chemical removing (by the solution based on composition KJ) of open metal areas through the previously obtained mask; and finally, (v) the vacuum deposition of the top metal film.

**Fig. 2:**
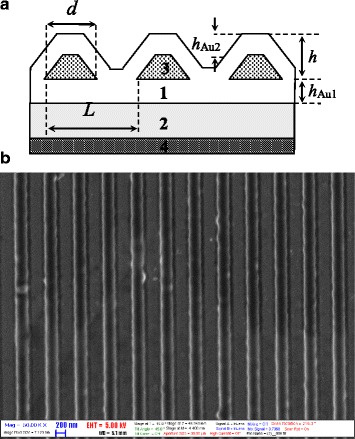
**a** Schematic cross section of heterostructure with quasi-anticorrelated interfaces of the metal film, where *1* semiconductor substrate; *2* *bottom* metal film with thickness *h*_*Au*__1_; *3* chalcogenide nanowires with width of basis, *d*; *3* metal *top* film with thickness *h*_*Au*__2_; and *4* ohmic contact. *L* is the structures period, and *h* is the grating depth. **b** SEM image of heterostructure surface fabricated by presented method based on the photoresistive laser interference lithography

Figure 2 a demonstrates the schematic cross-section of the surface barrier heterostructure “Au[ChP]Au/GaAs” where the “metal/semiconductor” interface is flat and the “metal/environment” interface possesses 1D periodical profiling. As we can see, the periodic inclusions of ChP wires into plasmon-carrying layer form a quasi-anticorrelation between the top interface of the frontal plasmon-carrying layer with a periodic profiling and bottom flat interface at Schottky barrier (the “metal/semiconductor” interface). Moreover, it is very important that in this case, the interface “metal/semiconductor” stills flat to avoid additional surface recombination losses in Schottky barrier and the interface “metal/environment” possesses a planar periodicity to support resonant condition for SPP excitation. The SEM image of structure surface is presented in Fig. 2 b and demonstrates uniformity of the relief over a large pattern area.

The period, *L*, of the structure under consideration is determined by parameters of the holographic setup. The grating depths, *h*, the width of basis, *d*, and cross-sectional shape of the chalcogenide wires are determined by the exposure and etching times. In our case, the corrugated interface has the form of a periodic array of reefs on a flat film, and it is appreciated by an isosceles trapezoidal cross-sectional shape with the trapeze ratio ranging from 0 (triangle cross section) to 1 (rectangular one). Here, the trapeze ratio is the ratio between small and large lengths of trapeze parallel sides. Changing these parameters, as well as the thickness of metal films *h*_*Au*__1_ and *h*_*Au*__2_, gives opportunities for optimization of optical parameters such quasi-anticorrelated structure.

The optimum value of the planar period is 750 nm in the case of the surface-barrier heterostructures under consideration based on GaAs. Other parameters are the following: the thickness of the lower metal film (*h*_*Au*__1_) is 20 nm; the thickness of the upper metal film (*h*_*Au*__2_) is 30 nm; and the width and height of ChP wire are 350 nm and 30 nm, respectively. This parameters are obtained during numerical optimization to achieve maximum polarization sensitivity (*T*_*p*_/*T*_*s*_), and they are used for all numerical simulation in this paper. The optical response of the “Au[ChP]Au/GaAs” heterostructure is compared with the equivalent “Au/GaAs” heterostructure with an optimal gold film thickness of 40 nm, where the 1D planar periodicity is formed by sinusoidal profiling of correlated metal film interfaces. This type of profiling of correlated metal film interfaces is typical in the case of conventional interference lithography with chemical etching of the semiconductor substrate.

For comparison of experimental and simulation results, we use measured photocurrent ratios with similar calculated transmittance ratios while the photocurrent is approximately proportional to the light transmittance.

## Results and Discussion

**Fig. 3:**
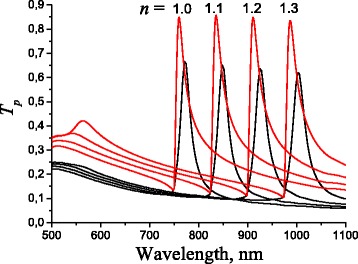
Spectra of *p*-polarized light transmittance at normal incidence for multilayer structure depending on the environment refractive index for heterostructures with quasi-anticorrelated (*trapezoidal cross-sectional shape* of ChP wire with a trapeze ratio of 0.2) and correlated interfaces of metal film (*sinusoidal* profile with a film thickness of 40 nm). Period 750 nm and grating depth 60 nm are the same for both structures

From the Eq. (3), it follows that the SPP signal sensitivity on the environment refractive index in the case of sensors based on periodically corrugated thin metal films is determined by two main factors: *∂* *λ*/*∂* *n* and *∂* *T*/*∂* *λ*. To analyze the influence of the correlation between both interfaces of plasmon-carrying layer on *∂* *λ*/*∂* *n*, the calculated transmittance spectra of *p*-polarized normal incident light for the optimized multilayer surface-barrier heterostructure “Au[ChP]Au/GaAs” with quasi-anticorrelated 1D periodically profiled interfaces and the equivalent structure “Au/GaAs” with correlated sinusoidal profiled metal film interfaces are shown in Fig. 3 for different refractive indices of the environment. These transmittance spectra demonstrate clearly observable peaks corresponding to the excitation of SPP. Moreover, the displacement of the SPP position due to the environment refractive index is practically independent of the correlation between both interfaces of plasmon-carrying layer. The extracted value of *∂* *λ*/*∂* *n* from Fig. 3 gives 760 nm/RIU.

**Fig. 4:**
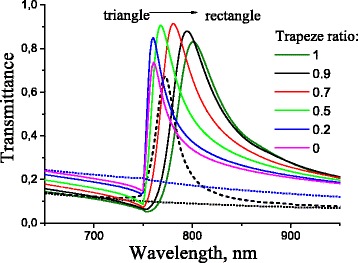
Spectra of *s*- and *p*-polarized light transmittance at normal incidence for multilayer structure with quasi-anticorrelated interfaces of metal film for various *shapes* of chalcogenide wires cross-section (changing from *triangular* to *rectangular shapes*). *Dashed line* corresponds to the *p*-polarized transmittance for correlated interfaces of metal film with sinusoidal profiles. *Dotted lines* correspond to the *s*-polarized transmittance for multilayer structure with correlated and quasi-anticorrelated interfaces of metal film. Structure parameters are the same as in Fig. 3

To analyze the impact of the reef shape on signal intensity, the calculated transmittance spectra for *p*-polarized light at normal incidence for the multilayer surface-barrier heterostructure “Au[ChP]Au/GaAs” with quasi-anticorrelated metal film interfaces for various cross-sectional shapes of ChP wires are presented in Fig. 4. The reef shape is varied from a triangular to a rectangular shape by changing the trapeze ratio between 0 and 1. The spectrum of *s*-polarized transmittance for the optimal reef shape (the trapeze ratio = 0.2) in the sense of the maximum value of the peak slope and the corresponding transmittance spectra for both polarizations in the case of the equivalent structure with correlated metal film interfaces with sinusoidal profiles are also added in Fig. 4. The transformation of Lorenz shape of resonant peaks in the case of correlated metal film interfaces with sinusoidal profiles (black line) into Fano shape in the case of quasi-anticorrelated interfaces is observed in Figs. 3 and 4. As a result, the slope of the resonance peaks is dramatically increased. Thus, the value of *∂* *T*/*∂* *λ* essentially depends on the resonance shape, and therefore the signal intensity sensitivity on the dielectric environment refractive index (Eq. (3)) can be enhanced by the proper design of the reef shape in the case of heterostructures with quasi-anticorrelated metal film interfaces.

**Fig. 5:**
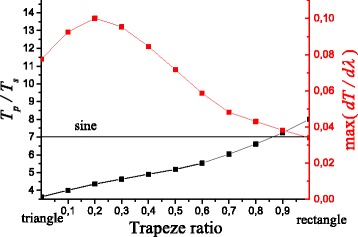
The dependence of polarization sensitivity (*T*_*p*_/*T*_*s*_) and signal intensity sensitivity on the wavelength (*∂* *T*/*∂* *λ*) for multilayer structures with quasi-anticorrelated metal film interfaces as a function of the *cross-sectional shape* of ChP wires (changing from *triangular* to *rectangular shape*). The *horizontal line* indicates the reference value for the equivalent structure with correlated metal film interfaces with sinusoidal profiles. Structure parameters are the same as in Fig. 3

Summarizing, the sensor parameters (the polarization sensitivity (*T*_*p*_/*T*_*s*_) and the signal intensity sensitivity on the wavelength (*∂* *T*/*∂* *λ*)) are extracted from Fig. 4 and presented in Fig. 5 depending on the trapeze ratio. It follows from Fig. 5 that the polarization sensitivity is increased more than two times for structures with quasi-anticorrelated metal film interfaces when the reef shape is changed from triangular (*T*_*p*_/*T*_*s*_=3.6) to rectangular (*T*_*p*_/*T*_*s*_=8) intersection. The polarization sensitivity is slightly smaller for an equivalent structure with correlated metal film interfaces with sinusoidal profiling (*T*_*p*_/*T*_*s*_=7). There is an opposite tendency for *∂* *T*/*∂* *λ*, when this value is reduced in the case of the reef shape transformation from a triangular to a rectangular cross section. *∂* *T*/*∂* *λ* reaches the maximum value (0.1 nm ^−1^) in the case of trapezoidal reef shape with the trapeze ratio equal to 0.2, that is, three times larger than the corresponding value for the equivalent structure with correlated metal film interfaces (*∂* *T*/*∂* *λ*=0.034 nm ^−1^). Estimations of the maximum of the signal intensity sensitivity on the environment refractive index (*∂* *T*/*∂* *n*) from Eq. (3) give values of 76 RIU ^−1^ for quasi-anticorrelated and 25.8 RIU ^−1^ for correlated metal film interfaces.

**Fig. 6:**
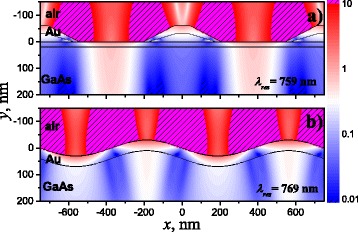
The spatial distribution of the electric field intensity *E* ^2^ for wavelength near SPP resonance in vicinity of plasmon-carrying layer with **a** quasi-anticorrelated interfaces (with trapezoidal ChP wires inclusions) and **b** correlated interfaces (sinusoidal profiling of a metal film interfaces). The *p*-polarized light is normally incident on the *top* of structures. Structure parameters are the same as in Fig. 3

The spatial distribution of the electric field intensity *E* ^2^ for the wavelength (*λ*_*res*_) near the SPP resonance is shown in Fig. 6 for two types of metal films: (a) quasi-anticorrelated interfaces of metal film formed by the inclusions of ChP wires with trapezoidal cross-sectional shape (“Au[ChP]Au/GaAs” heterostructure) and (b) the equivalent structure with correlated sinusoidal profiled interfaces of metal film. The electric field intensity *E* ^2^ is normalized on the intensity of incident plane wave. As we can see from a comparison of the spatial distributions of the electric field intensities in Fig. 6, the light mostly penetrates into the semiconductor active region through a narrow plane-parallel parts of a plasmon-carrying layer in the case of quasi-anticorrelated interfaces with ChP wire inclusions, while the light penetrates absorptive plasmon-carrying layer from upper reefs in the case of sinusoidal profiled corrugated metal film. It should be noted that the light reflection is reduced at upper side of trapezoidal reefs in the case of quasi-anticorrelated metal film interfaces with ChP wire inclusions that is typical for the localized modes. This means that we observe two modes of different nature in Fig. 6 a, which leads to the formation of the optical response with the Fano shape of peaks: (i) continuous SPP waves that are responsible for high resonant transmission through the metal film and (ii) localized modes which are responsible for the light absorption in the region of reefs (that means that the part of the reflected light energy is reduced).

**Fig. 7:**
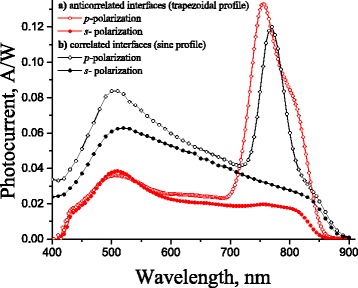
Spectral dependence of the photocurrent measured for *p*- and *s*-polarized light at normal incidence for two types of metal film correlation in surface-barrier heterosrtuctures based on Schottky barrier with geometrical parameters: **a** quasi-anticorrelated relief *L*=750 nm, *h*=50 nm, *h*_*Au*__1_=20 nm, and *h*_*Au*__2_=35 nm and **b** correlated (sinusoidal) relief *L*=760 nm, *h*=70 nm, and *h*_*Au*_=40 nm. The schematic cross section of structure with quasi-anticorrelated metal film interfaces is presented in Fig. 2

The experimental spectral dependences of the photocurrent induced by the *s*- and *p*-polarized light at normal incidence for the surface-barrier heterostructures with quasi-anticorrelated and correlated interfaces of a thin metal film forming Schottky barrier at the “metal/semiconductor” interface are presented in Fig. 7. As we can see, the resonance maxima are observed in both structures under consideration in the wavelength range between 720 nm and 800 nm for the *p*-polarized light in the case of both structures with different interface correlations. These photocurrent peaks are associated with SPP excitation at the 1D periodically profiled “metal/environment” interface in accordance with the definition of SPP excitation in the Eq. (1). In addition, the polarization sensitivity increases to 6.75 at a wavelength of 755 nm due to increasing of the light transmittance into the active region of a semiconductor in the case of the surface barrier heterostructure with quasi-anticorrelated metal film interfaces, while the polarization sensitivity is 3.9 at 770 nm in the case of correlated metal film interfaces with sinusoidal profiling. Maximum value of *∂* *T*/*∂* *λ* = 0.0033 (A/W) nm ^−1^ was obtained in the case of a trapezoidal shape that can be associated with simulation data for a trapeze ratio of 0.8, and 0.0029 (A/W) nm ^−1^ for a sinusoidal shape.

## Conclusions

The surface plasmon-polariton photodetectors based on diffraction gratings with quasi-anticorrelated interfaces of the metal film have been proposed for the first time. This grating formation approach based on laser interference lithography technology without the etching of the semiconductor substrate. Moreover, this manufacturing process is relatively simple and more compatible with microelectronic technological processing.

In the framework of the differential formalism with assumption that all media forming multilayer structures with curvilinear interfaces are homogeneous with bulk optical constants, it has been shown theoretically that the polarization sensitivity strongly depends on the cross-sectional shape of chalcogenide wires and increases more than two times in the case of transition from a triangular shape of wire crossing (*T*_*p*_/*T*_*s*_=3.6) to a rectangular one (*T*_*p*_/*T*_*s*_=8). The value of *T*_*p*_/*T*_*s*_ is 7 for an equivalent structure with correlated gold film interfaces with sinusoidal profiles. It was found that the signal intensity sensitivity on the wavelength (*∂* *T*/*∂* *λ*) increases relatively to the equivalent structure with correlated metal film interfaces with sinusoidal profiles if the cross-sectional shape of chalcogenide wires has an isosceles trapezoidal shape. Maximum value of *∂* *T*/*∂* *λ*=0.1 nm ^−1^ was obtained in the case of an isosceles trapezoidal shape with the ratio between small and large lengths of the trapezium parallel sides equals to 0.2 that is three times larger than for an equivalent structure with correlated metal film interfaces with sinusoidal profiles with *∂* *T*/*∂* *λ*=0.034 nm ^−1^. Corresponding estimations of the maximum of the signal intensity sensitivity on the environment refractive index (*∂* *T*/*∂* *n*) give 76 RIU ^−1^ for quasi-anticorrelated and 25.8 RIU ^−1^ for correlated metal film interfaces.

Theoretical predictions were confirmed experimentally by comparing the spectra of the photocurrent for the surface barrier heterostructures based on Schottky barrier with quasi-anticorrelated and correlated interfaces of the metal film.
